# AFLW and the gender gap: an analysis of public attitudes towards the Women’s Australian Football League

**DOI:** 10.1080/00049530.2024.2315949

**Published:** 2024-02-12

**Authors:** Mackenzie Rose Glazbrook, Stephanie Newton Webb

**Affiliations:** Justice and Society, University of South Australia, Adelaide, Australia

**Keywords:** Women’s sport, attitudes, gender roles, prejudice, sports consumerism

## Abstract

**Objective:**

The introduction of the Women’s Australian Football League (AFLW) has highlighted the inequality faced by women football players (e.g., season length), with the consumerism and fanbase of the women’s AFL lacking comparatively with the men’s league. The paucity of research into the AFLW and attitudes towards women’s sport, limits our understanding of the predictors of these attitudes. Therefore, the primary aim of this study was to investigate the factors that may influence the development of attitudes towards the AFLW.

**Method:**

Data were collected from a community sample (*N* = 171), to evaluate attitudes towards both AFLW consumerism, and the endorsement of gender equality within the AFL.

**Results:**

Results revealed that basic demographic factors were influential in attitude development (i.e., age, gender), however the most prominent predictor of attitudes towards the AFLW was gender role ideology, suggesting that beliefs about gender roles may influence anti-AFLW attitudes.

**Conclusions:**

The findings reflect an ongoing perception of sport as a masculine space, and as such, a need to adjust marketing approaches to frame the AFLW in a manner that encourages consumer behaviour. Future research directions are discussed.

## KEY POINTS

**What is already known about this topic:**

1. Women’s participation in sport, particularly traditionally masculine sport, has historically been stigmatised.
2. Women’s sports receive less consumer engagement than men’s sports, impacting the financially sustainability of women’s sporting leagues.
3. Gender role ideology influences the way people perceive the suitability of individual participation in various activities within a societal context.

**What this topic adds:**

1. The current study provides insight into the factors that may influence consumer attitudes surrounding the recently established Women’s Australian Football League.
2. Traditionalist gender role ideals influence negative attitudes towards both consumerism of women’s football, and endorsement of gender equality within football. That is, those with a traditional understanding of gender are less likely to consume women’s football and are more likely to endorse perceptions that men’s football should take precedence.
3. Attempts to encourage positive consumer behaviour for women’s football in Australia require intervening in perceptions of football as a sport that is predominantly suited to men.

Despite the progress towards gender equality in Australian society, the sporting industry has maintained its aura of male domination. Historically, women have been enthusiastic consumers and participants of sport in Australia, although they have seldom been considered competent athletes (Willson et al., 2017). This may be due to a firmly held belief that certain sports are inappropriate for women (Fink, 2016). Sport, as an institution, encompasses potentially the most traditionally gendered disposition present in contemporary society (Schull & Kihl, 2019). Its innate support of patriarchal values allows for the celebration of traits that are traditionally credited to men, permitting the exclusion of women athletes (Schull & Kihl, 2019), particularly in sports dominated by men. This rhetoric is demonstrated by research suggesting that women are often considered inferior athletically, as such, participation in sports that are deemed feminine is considered more socially acceptable (Pfister, 2010). These attitudes appear to be reinforced by perceptions of the traditionally sectored nature of socially constructed gender roles (Pfister, 2010). For example, historically, individuals have attributed the lack of women’s participation in sport to apparent physical and biological differences between women and men, assuming that men are more capable athletes (Fink et al., 2016). However, research has shown that biological sex may predict only limited differences in physical ability (Chalabaev et al., 2013), suggesting that the discrepancy in popularity of sports played between women and men, may come from misunderstandings and sexist attitudes. Therefore, the aim of the current study is to investigate the potential factors that may be influential in the development of attitudes towards women’s participation in a popular male-dominated sport, Australian rules football.

## Australian rules football

Australian rules football (which from hereon will be referred to simply as football) is a physically demanding, high contact sport that is unique to Australia. The first officially recognised (men’s) football match was played in 1858, with popularity for the sport increasing greatly over time (Nicholson et al., 2021). Despite being the most renowned sporting competition in Australia (Willson et al., 2017), the football industry has failed, until recently (i.e., 2017), to include women at a professional level. Historically, football has been considered a traditionally masculine sport, despite women’s participation in the early 1900s (Willson et al., 2017), evident in the exclusion of women, professionally. However, recent research has demonstrated an increase in women’s participation in football in Australia (e.g., 790% in the past decade), leading to the eventual establishment of the Women’s Australian Football League (AFLW), as an equivalent branch of the Australian Football League (men’s) (Willson et al., 2017). The introduction of the AFLW has had an influence on the social and cultural dynamics of Australian football, by challenging previously established social norms in this industry, that suggest football is only for men. Despite the positive impact of the AFLW, its establishment has also highlighted a significant gap in equality between the women’s and men’s leagues, potentially hindering successful development of the league.

### Inequalities within the Australian football leagues

The introduction of the AFLW, although a progressive step forward in the fight for gender equality, was not without both compromise and disadvantage, and notable differences between the competitions (i.e., women’s and men’s (AFLM)). Firstly, the AFLW runs a shorter season of 10 rounds, which does not allow all teams to play each other, ultimately leading to an arguably less fair competition (Australian Football League [AFL], 2023). Further, despite football traditionally being a winter sport, due to the high intensity of athletic needs during the game, the AFLW are required to endure Australia’s hotter seasons to avoid their competition overlapping with the AFLM (Willson et al., 2017). Additionally, AFLW teams play with 16 players per team instead of the usual 18, and match quarters have a duration of 17-minutes rather than the usual 20-minutes (AFL, 2023). Moreover, AFLW matches are predominantly played at local and state level ovals, as opposed to the nationally recognised stadiums at which the AFLM competition exhibits games, limiting AFLW players’ access to quality training facilities and potential space for spectators. These disparities reflect the state of international sports (e.g., WNBA and NBA), in which men’s leagues take precedence (Walker et al., 2012). Such discrepancies may reinforce the perception that AFLM is the superior product, therefore limiting consumer motivation to engage with the AFLW.

Furthermore, football is no exception in reference to the gender-wage gap. There have been significant differences in pay between the AFLW and the AFLM, with several AFLM players earning over $1 million per year, whereas the highest paid AFLW players have comparatively earned only $72, 000 per year (an increase from only $30,000 only two years prior; The West Australian, 2020). This wage gap often sees AFLW players needing a second source of income to support themselves. Meaning players are forced to decide between pursuing their primary career or their football career; a decision that AFLM players do not face (Willson et al., 2017). Whilst the announcement of the 2023 Collective Bargaining agreement confirmed an additional pay increase for the AFLW, the average wage of women players remains at just 16% of the average wage of men players (Waterworth, 2023). Although a problematic issue, it is pertinent to emphasise the differences in consumption and investment in men’s sports more generally that may facilitate this wage gap, reflecting the imbalance in consumer interest between women’s and men’s football more broadly. Given the history in Australia for the fight for wage equality within sports that host women and men leagues (e.g., soccer) spanning over more than 40 years, the gender-wage gap present in football cannot be entirely attributed to the newness of the AFLW, suggesting it may stem from, not only the patriarchal stereotypes embedded within the profession, but also issues within consumer attitudes. Therefore, highlighting the need for the current research to develop a foundation for understanding what factors may predict attitudes towards the AFLW.

### Attitudes towards women in sport and the AFL

#### Sports consumerism

Given the importance of consumers in contributing to revenue, issues surrounding the financial sustainability of women’s professional sport often stem from limited popularity and negative attitudes (Mumcu & Marley, 2017). Previous research has shown that attitudes are strong predictors of observable behaviour (Pradhan et al., 2020) and strong attitudes have been shown to motivate positive consumer behaviour (Funk et al., 2002). Consumer behaviour refers to an individual’s intention to invest money into a product (including inadvertently), based on their own desires and interest (Ajzen, 2008). In the context of sport, this may include attending matches, buying merchandise, and watching games digitally. Motivational factors that may contribute to attitudes towards sports consumerism include entertainment value, social interaction, aesthetic, achievement and both actual and perceived physical skills of athletes (Mumcu & Marley, 2017). For example, when the skills of athletes are perceived as high quality, this equates to increased entertainment and, therefore, attitudes towards the sport or competition are more favourable (Mumcu & Marley, 2017). Alternatively, if the skills of athletes are perceived as being of poor quality, people may exhibit less favourable attitudes, and therefore, are less likely to support or engage with the competition. Although there is considerable research into the relationship between attitudes of sports and behavioural outcomes, much of this is on men’s sport, limiting our understanding of attitudes towards and consumerism of women’s sport. As previous research has emphasised the factors that encourage women’s sports consumerism likely differ to the factors that may encourage to men’s sports consumerism (see for example Kim et al., 2019; Thomson et al., 2023), this topic remains an important area of academic enquiry.

#### Demographic and socio-demographic factors

The literature in this area has demonstrated some demographic and socio-demographic factors that may predict attitudes towards women in sport. For example, research has found that men (Harry, 1995; Mumcu & Marley, 2017), former competitive athletes, and older adults (Mumcu et al., 2016) are more likely to endorse negative attitudes towards women’s sport. Furthermore, there is substantial research that emphasises the mediating role of knowledge on attitude formation, and the notion that more extensive knowledge may equate to stronger positive attitudes (McPhetres et al., 2019; Weisberg et al., 2020). Research has also demonstrated that individuals who hold prejudicial attitudes towards sexual minorities (Lee & Cunningham, 2016) have historically been found to be less likely to endorse positive attitudes towards women in sport (Harry, 1995). This may be due to a common stereotype that women who play *masculine* sports are assumed to be part of a sexual minority group (Scheadler & Wagstaff, 2018), and may indicate a relationship between negative attitudes towards the AFLW and anti-sexual minority ideals.

##### The influence of gender role ideology

Gender role ideology refers to the beliefs or attitudes individuals hold about the perceived or expected roles of women and men in a societal context (Somech & Drach-Zahavy, 2016). The variance in participation and inclusion in sport between women and men may largely be attributed to the barriers created by societal perceptions of gender roles (Pfister, 2010). *Social Role Theory* (SRT) purports that behavioural differences between women and men emerge from societal stereotypes of gender, which are thought to form from a phenomenon known as *Correspondence Bias*: the “cognitive process of inferring traits from observed behaviour” (Eagly & Wood, 2012, p. 462). That is, social perceivers typically interpret an individual’s behaviour as a reflection of their innate biological characteristics, rather than a consequence of cultural and environmental influences. This effect is demonstrated by gender roles that emerged from the division in labour and the division of power (i.e., gender hierarchy) present, predominantly, in Western societies. Historically, women and men have been allocated tasks on the basis of their physical attributes (e.g., women were more likely to fulfil tasks related to children and family, whereas men were often allocated tasks that required strength, stamina, and authority; Eagly & Wood, 2012). As such, traditional gender roles refer to beliefs adhering to a traditional gender binary in which tasks are separated on the basis of gender. In contrast, egalitarian gender ideologies generally promote equality through the belief that tasks, and characteristics are not gender specific (Soltanpanah et al., 2017). In contemporary society there has been a shift towards egalitarian ideals, as emphasis on assigned gender roles has weakened as a consequence of the decreased importance of physical sex differences due to (a) lower birth-rates, and (b) reduced reliance on size and strength as requirements for competency in the workforce, and (c) a societal shift against gender inequality (Eagly & Wood, 2012). However, women are still less likely to be appointed roles that are deemed traditionally masculine, reinforcing perceptions of what is considered appropriate gendered behaviour, therefore, retaining some degree of patriarchy in contemporary society (Eagly & Wood, 2012).

Sport has historically been considered a *masculine* activity, as stereotypically masculine traits such as strength, speed, and stamina are often required for success – assumed to only be possessed by men. The exclusion of women from traditionally *masculine* sports may both contribute to, and act as a result of societal gender roles (Chalabaev et al., 2013). That is, the lack of women’s participation in masculine sports reinforces the stereotype that women are biologically not suited to participate (Gentile et al., 2018). When women do enter into sport that is traditionally masculine, it challenges perceptions of these gender roles, blurring the lines of the gender dichotomies that many people assume exist (McCabe, 2007; Stewart et al., 2021), which may create feelings of discomfort for those who endorse a traditional binary of gender roles. Past research supports this by demonstrating that those with more traditional gender ideology respond more negatively to those who violate traditional gender norms, as it disturbs their need to sustain the perception of gender as binary construct (McCabe, 2007), whilst individuals with egalitarian ideals of gender roles have been found to possess more positive attitudes towards women in sport (McCabe, 2007). Given that football has historically been considered a masculine sport, women’s participation may be seen as a violation of societal gender norms that threatens patriarchal values of the role of women (Schull & Kihl, 2019). Therefore, traditionalist ideals of gender may predict negative attitudes towards the AFLW; however, a dearth of research exists on the factors that influence attitudes towards Australian football.

##### Sexualisation of women athletes

The emphasis placed on the need for women athletes to be feminine and sexually attractive to be considered marketable, may also contribute to the trivialisation of women in sport, particularly in male dominated sports (Pfister, 2010). Past research demonstrates that media reports on male athletes primarily comment on their athletic performance, whilst media reports on women athletes are often found to emphasise factors irrelevant to their sporting performance, such as sexual attractiveness (Fink, 2015). This serves to maintain the perception of women’s sport as a novelty, whilst undermining women athletes’ contribution to the sporting industry. Furthermore, a study grounded in objectification theory, found that attitudes towards women athletes who have been objectified by the media, which is disproportionately more common for women, are often less favourable than those towards women athletes who are known for their performance (Daniels et al., 2020). This same objectification of women is found within the AFLW. Perhaps the most infamous example was the incident involving AFLW player, Tayla Harris in 2019. A photograph of Harris was posted to social media, depicting her with her legs split open (i.e., the necessary motion when kicking a football). This post was flooded with derogatory, sexist comments that belittled Harris and her athletic ability (e.g., the photo was edited to make Harris appear naked in a compromised position; Marshall, 2021); comments that would not be made towards men for performing the same action. Since this incident, Harris has consistently been victim to online “trolls” and harassment (Marshall, 2021), which coincides with the research from Daniels et al. (2020), suggesting that the sexual objectification of women athletes may also predict negative attitudes towards the AFLW.

### The current study

Due to the recency of its establishment, there is limited research into the AFLW and consumer behaviour surrounding women’s football in Australia. Existing research of similar domains has been utilised to suggest that several factors may be important in understanding attitudes towards women in sport. In conjunction with a review of the relevant literature, the aim of the present study is to investigate two variations of public attitudes towards the AFLW: (1) attitudes towards AFLW consumerism, and (2) attitudes towards the endorsement of gender equality within the AFL. The current research is important as it will provide knowledge that can be used to identify and combat factors that lead to imbalanced consumerism and poorer attitudes towards the AFLW. This may benefit the football industry, due to the sporting industry’s heavy reliance on consumers for financial sustainability. This study will address the following research question:

RQ1: *What are the key factors predicting attitudes toward the AFLW?*

Additionally, the following research hypotheses will be tested:

H1: It is expected that there will be a significant positive relationship between gender and attitudes towards the AFLW. That is, women will endorse more positive attitudes towards the AFLW than men.

H2: It is expected that there will be a significant negative relationship between age and attitudes towards AFLW. That is, older participants will endorse more negative attitudes towards the AFLW than younger participants.

H3: It is expected that there will be a significant negative relationship between gender role ideology and attitudes towards the AFLW. That is, individuals with traditional ideals of gender roles will have less favourable attitudes towards the AFLW than those with egalitarian ideals of gender roles.

H4: It is expected that there will be a significant positive relationship between knowledge and attitudes. That is, participants with more knowledge of AFLW, will have more positive attitudes towards the league than those with limited knowledge of AFLW.

H5: It is expected that participants’ attitudes towards gender roles and sexualisation of women will predict attitudes preferencing equality within football, over and above all other factors measured.

H6: It is expected that participants’ attitudes towards gender roles and sexualisation of women will predict attitudes towards AFLW consumerism, over and above all other factors measured.

## Method

### Sample

A community sample of 357 participants responded to an online survey, administered via the platform *SurveyMonkey* in 2021. Participants were recruited from social media platforms (i.e., Facebook, Instagram) where the survey link was provided. Snowballing methods were implemented to increase participation, whereby participants were asked to share the survey link with others. Ethical approval was granted by the host University (protocol #203788).

Of the initial 357 participants, 183 completed an insufficient number of survey items (e.g., incomplete scales) and thus were deleted from the sample. Two participants identified themselves as non-binary or transgender and were excluded from the final data file due to the small sample size. The final sample of 171 participants were comprised of 123 women (71.1%) and 48 men (27.7%). Ages ranged from 18 to 70, and a majority (96%) of participants resided in South Australia (see Table 1 for a breakdown on the demographic participant information).

**Table 1.** Demographic characteristics of the final sample.

Variable	Mean	SD	% of N	N
Age (range 18–70)	38.57	14.81		171
Gender				
Men			27.7	48
Women			71.1	123
Sexual Orientation				
Heterosexual			89.6	155
Sexual Minorities^a^			10.4	18
AFL Experience				
Current/Previous Player			40.5	70
Non-player			59.5	103
AFL Fandom				
AFLM Followers			79.2	137
AFLW Followers			43.4	75

^a^Sexual Minorities include bisexual, homosexual, and pansexual.

### Measures

The scales are described below in the order that they were presented online to participants. Each participant was shown an identical format of the questionnaire. All measures utilised the same 6-point forced-response scale (1 = strongly disagree; 6 = strongly agree), unless otherwise indicated.

#### Demographic information

Data on participant age (in years), gender, sexual orientation, location of residence, level of AFL experience (e.g., amateur, state league, professional level), and level of support for AFL (e.g., “Do you currently follow AFLW/AFLM?”) were collected.

#### AFLW exposure

Three single-item indicators were used to assess participants’ exposure of the AFLW (“Have you ever watched an AFLW match on television?”, “Have you ever attended an AFLW match?”, “Do you follow any AFLW related social media?”). All items were dichotomous. These items were averaged to create a score between 1–6, with higher scores indicating greater exposure of AFLW.

#### AFLW consumption attitudes

A modified version of the 34-item Attitudes Towards Women’s Sports Scale (ATWSS; Mumcu, 2013) was utilised to assess AFLW consumption attitudes. The ATWSS consists of eight subscales: excitement (4-items, e.g., “AFLW matches are exciting”), opportunity for women (4-items, e.g., “the AFLW increases opportunities for women in life”), accessibility (4-items, e.g., “it is easy to find information about the AFLW), aesthetic (5-items, e.g., “AFLW matches are a form of art”), affect (5 scale items), drama (4-items, e.g., “AFLW matches are usually close games”), entertainment value (3-items, e.g., “AFLW matches are expensive [reverse coded]), and athlete quality (5-items, e.g., “AFLW players have excellent skills”). Higher scores on the ATWSS indicated more positive attitudes towards consumerism of women’s football (maximum possible score = 204). Reliability analyses demonstrated excellent internal consistency for the ATWSS (α = .95).

#### Attitudes towards gender equality within the AFL

A 10-item scale was constructed to assess attitudes towards the endorsement of gender equality within the AFL (e.g., “women should not have a place in professional football” [reverse scored]). This scale was created from common discourse around the inequalities between the leagues (Willson et al., 2017) and some modified items from the Attitudes of Sports Media towards Women’s Sports scale (Menevşe & Emin Ablay, 2019). Higher scores indicated more positive attitudes towards gender equality within football (maximum possible score = 60). Reliability analyses revealed good internal consistency (α = .80) in this sample.

#### Attitudes towards gender roles

The 13-item Social Roles Questionnaire (SRQ; Baber & Tucker, 2006) was used to assess attitudes towards perceived societal gender roles. This questionnaire includes two subscales: Gender-Linked (GL) (8-items) and Gender Transcendence (GT) (5-items). The GL subscale assesses perceptions of whether gender is associated with specific societal roles (e.g., “only some types of work are appropriate for both men and women”). The GT subscale assesses the degree to which gender is perceived in a non-traditional way (e.g., “tasks around the house should not be assigned by sex”). Higher scores indicated more traditional gender role beliefs (maximum possible score = 78). For the purpose of this study, the total scale was used, demonstrating good internal consistency (α = .85) in this sample.

#### Sexualisation of women

Attitudes surrounding the sexualisation of women were assessed using the 8-item “Women are Sexual Objects” subscale, taken from the Attitudes Toward Dating and Relationship Measure (Ward, 2002) (e.g., “there’s nothing wrong with men whistling at shapely women”). Higher scores indicated a more sexualised view of women (maximum possible score = 48). This measure demonstrated acceptable internal consistency (α = .76) for this sample.

#### Attitudes towards sexual minorities

The 7-item Homophobia Scale (Roese et al., 1992) was used to assess attitudes towards sexual minorities (e.g., “homosexual behaviour disgusts me”). Higher scores on this measure indicated more negative attitudes towards sexual minorities (maximum possible score = 42). This measure demonstrated acceptable internal consistency (α = .74) for this sample.

#### Knowledge of AFLW and AFLM

A 12-item quiz was constructed to assess participants’ knowledge of AFL (e.g., “How many teams are there in the AFLW/AFLM?”). Six items measured knowledge of the AFLW and created an AFLW knowledge subscale, and 6 items assessed knowledge of AFLM and created an AFLM knowledge subscale. Correct items were sourced from up-to-date information of the AFLW and AFLM (AFL, 2021c; AFLW, 2021; McFarlane et al., 2021). All items used a multiple-choice format. Items were scored dichotomously (correct = 1, or incorrect = 0), with scores ranging from 0–6 for AFLW and AFLM, respectively. Higher scores indicated more knowledge of AFLW/AFLM. This measure demonstrated acceptable internal consistency (α = .72) in this sample.

### Data analysis

Data were exported from *SurveyMonkey* to IBM SPSS v.28, cleaned, and screened prior to analysis. Bivariate correlations were conducted to assess the relationships between the predictor variables of interest and the outcome variables. Two hierarchical regressions were utilised to test whether gender role ideology, and the sexualisation of women uniquely predicted attitudes towards the AFLW. The assumptions of hierarchical regression (i.e., homoscedasticity, linearity, and absence of multicollinearity) were all met. Scatterplots were used to assess homoscedasticity and linearity, whilst Variance Inflation Factors determined the presence of multicollinearity. The first hierarchical regression was conducted to assess the relationship between the predictor variables and attitudes towards endorsement of gender equality within football (outcome variable). The second hierarchical regression was conducted to assess the relationship between the predictor variables and AFLW consumption attitudes (outcome variable). In each regression, demographic variables were controlled for and entered at step one. Sport specific variables were entered at step two. The main predictor variables were entered at the final step (gender role attitudes, and sexualisation of women).

## Results

### Preliminary analyses

#### Attitudes towards the endorsement of gender equality within the AFL

Bivariate correlations revealed significant negative relationships between gender [H1 – confirmed], age [H2 – confirmed], sexual orientation, gender role ideology [H3 – confirmed], sexual minority prejudice, and sexualisation of women with attitudes towards gender equality within football. That is, older participants, men, those who held more traditional gender role beliefs, those who expressed more sexual minority prejudice, those who were heterosexual, and those more likely to sexualise women, all held significantly poorer attitudes towards the endorsement of gender equality within the AFL. Results also revealed a significant negative relationship between AFLM knowledge and attitudes towards equality within the AFL. That is, as knowledge of AFLM increased, positive attitudes decreased. Results revealed AFLW knowledge and AFLW exposure to be non-significant and these were therefore excluded from the final analysis (see Table 2 for all correlation coefficients).

**Table 2.** Correlation matrix of significant predictors of attitudes towards consumerism of the Women’s Australian football league (ATWSS), and attitudes towards gender equality within Australian rules football.

Predictor	2	3	4	5	6	7	8	9	10	11	12	13	14	Mean (SD)
Age	.242**	.084	.344**	−.071	−.143	−.017	−.050	.021	−.181*	−.011	−.081	−.156*	−.088	38.57 (14.81)
Gender	–	.022	−.627**	−.116	−.051	−.120	.338**	.150	.444**	.346**	.210**	−.231**	−.101	
Sexual Orientation		–	−.011	−.338**	.046	.065	.175*	−.033	.100	.231**	.049	−.373**	−.258**	
Football Experience			–	.161*	.253**	.348**	−.283**	−.240**	−.336**	−.232**	−.147	.156*	−.093	
AFLM Follower				–	.391**	.360**	−.561**	−.396**	−.055	−.121	−.150	.327**	.004	
AFLW Follower					–	.729**	−.311**	−.431**	.105	.132	−.017	−.154*	−.465**	
AFLW Exposure						–	−.393**	−.560**	.086	.021	−.017	−.027	−.432**	4.57 (1.00)
AFLM Knowledge							–	.532**	.012	.017	.027	−.231**	.035	3.59 (1.61)
AFLW Knowledge								–	−.029	−.074	.000	−.093	.155*	2.20 (1.52)
WSO^a^									–	.627**	.339**	−.440**	−.269**	16.17 (5.6)
SRQ^b^										–	.439**	−617**	−428**	23.97 (8.25)
HS^c^											–	−.203**	−.106	11.07 (4.81)
Equality Scale												–	.601**	42.86 (8.51)
ATWSS													–	154.15 (26.16)

^a^“Women are Sexual Objects” subscale (Ward, 2002), ^b^Social Roles Questionnaire (Baber & Tucker, 2006), ^c^Homophobia Scale (Roese et al., 1992).

*p < .05. **p < .01.

#### Attitudes towards AFLW consumerism

Bivariate correlation results revealed significant negative relationships between sexual orientation, AFLW exposure, sexualisation of women, and gender role attitudes [H3 - confirmed] with AFLW consumption attitudes (ATWSS). Furthermore, a significant positive relationship was found between AFLW knowledge and the outcome variable [H4 - confirmed]. That is, those who identified as part of a sexual minority, those who had been exposed to more AFLW, and those with more knowledge of AFLW were all more likely to hold positive AFLW consumerism attitudes. In contrast, those who held more traditional gender role beliefs, and those more likely to sexualise women, held more negative AFLW consumerism attitudes. Age [H2 – refuted], gender [H1 – refuted], football experience, AFLM followers, AFLM knowledge, and sexual minority prejudice were not found to be significantly correlated with this outcome variable and were therefore excluded from the final analysis (see Table 2 for all correlation coefficients).

### Main analyses

#### Attitudes towards the endorsement of gender equality within the AFL

A 3-step hierarchical multiple regression was conducted to assess the factors predicting attitudes towards gender equality within AFL. In step 1, age, gender, sexual orientation, and sexual minority prejudice were included and accounted for 20% of variance in attitudes towards the endorsement of gender equality within AFL. In step 2, sport specific factors (football experience, AFL support, and AFLM knowledge) were added and accounted for 34% of variance in these attitudes. In step 3, gender role attitudes, and sexualisation of women were added and accounted for an additional 20% of variance. The final model accounted for 54% of variance in attitudes towards the endorsement of gender equality within AFL. Both the sexualisation of women attitudes (ß = −.171, *p* < .05) and gender role attitudes (ß = −.434, *p* < .001) significantly predicted attitudes towards the endorsement of gender equality within the AFL, over and above the variance explained by other factors (*R*^*2*^ Δ = .199, *F* (2, 152) = 33.16, *p* < .001). Only age, AFLM followers, AFLW followers, gender role beliefs, and sexualisation of women remained significant in the final model. See Table 3 for hierarchical regression results.

**Table 3.** Hierarchical regression predicting attitudes towards the endorsement of gender equality within the AFL.

	Model 1			Model 2			Model 3		
Variables	*B*	*SE B*	β	*B*	*SE B*	β	*B*	*SE B*	β
Age	−.011	.004	−.187*	−.016	.004	−.272**	−.013	.004	−.229**
Gender	−.411	.139	−.220**	−.176	.168	−.094	.127	.148	.068
Sexual Orientation	−.943	.208	−.322**	−.541	.209	−.184*	−.305	.178	−.104
HS^a^	−.187	.089	−.152*	−.149	.084	−.121	.097	.076	.079
Footy Experience				.338	.164	.195*	.227	.138	.131
AFLM Follower				.592	.187	.277**	.504	.158	.236**
AFLW Follower				−.643	.131	−.376**	−.448	.113	−.263**
AFLM Knowledge				−.038	.045	−.072	−.052	.038	−.099
WSO^b^							−.026	.011	−.171*
SRQ^c^							−.045	.008	−.434**
*R*^*2*^	.195			.345			.544		
*F* Change	10.81			7.65			33.16		

*p < .05. **p < .01; β = standardised, *B = unstandardised*.

^a^Homophobia Scale (Roese et al., 1992), ^b^“Women are Sexual Objects” subscale (Ward, 2002), ^c^Social Roles Questionnaire (Baber & Tucker, 2006).

#### Attitudes towards AFLW consumerism

A 3-step hierarchical multiple regression was conducted to assess the factors predicting attitudes towards AFLW consumerism. In step 1, sexual orientation was included and accounted for 6% of variance in AFLW consumption attitudes. In step 2, sport specific factors (AFLW exposure, AFLW follower, and AFLW knowledge) were added and accounted for 29% of variance. In step 3, gender role attitudes, and sexualisation of women were added and accounted for an additional 12.5% of variance. The final model accounted for 41% of variance in AFLW consumerism attitudes. Gender role attitudes (ß = −.400, *p* < .001) significantly predicted AFLW consumerism attitudes, over and above the variance explained by other factors (*R*^*2*^ Δ = .125, *F* (2, 158) = 16.86, *p* < .001). Sexual orientation, AFLM followers, AFLW followers, AFLW knowledge, and gender role beliefs remained significant in the final model. See Table 4 for hierarchical regression results.

**Table 4.** Hierarchical regression predicting attitudes towards AFLW consumerism.

	Model 1			Model 2			Model 3		
Variables	*B*	*SE B*	β	*B*	*SE B*	β	*B*	*SE B*	β
Sexual Orientation	−.555	.173	−.244**	−.509	.152	−.224**	−.317	.143	−.139*
AFLW Follower				−.455	.137	−.336**	−.344	.127	−.246**
AFLW Exposure				−.170	.074	−.244*	−.212	.069	−.306**
AFLW Knowledge				−.059	.037	−.129	−.070	.034	−.154*
WSO^a^							.007	.010	.053
SRQ^b^							−.034	.007	−.400**
*R*^*2*^	.060			.287			.412		
*F* Change	10.33			16.97			16.86		

*p < .05. **p < .01; β = standardised, *B = unstandardised*.

^a^“Women are Sexual Objects” subscale (Ward, 2002), ^b^Social Roles Questionnaire (Baber & Tucker, 2006).

## Discussion

The purpose of the current study was to develop an understanding of the factors that may be important in the development of attitudes towards the AFLW. Two variations of attitudes were measured: (1) attitudes towards AFLW consumerism, and (2) attitudes towards the endorsement of gender equality within football. As expected, results revealed that gender role attitudes and attitudes towards the sexualisation of women predicted negative attitudes towards the endorsement of gender equality within the AFL, over and above all other factors, with gender role attitudes also significantly predicting AFLW consumerism attitudes over and above other factors.

The finding that both gender role attitudes and the sexualisation of women may play an important part in influencing attitudes towards women’s participation in the AFLW, feeds into a broader understanding of SRT in the context of Australian sport. According to SRT, individuals with traditional ideals of gender perceive gender as a dichotomous construct and maintain the premise that certain activities and roles are either feminine or masculine, and subsequently, either for women or men (Eagly & Wood, 2012). Due to the conventionally masculine nature of football (e.g., the requirement for *masculine* skills such as strength and speed), compared to other sports (e.g., tennis), those with traditional ideals of gender likely regard the AFLW as a violation of gender norms that act to rationalise the patriarchal dominance within football, and Australian society more generally, justifying why participants with more traditional gender ideology were less likely to endorse gender equality between the leagues. This indicates a belief that women’s football is not worthy of the same funding and resources provided to men’s football, which by extension, may suggest reluctance to personally invest in the league.

Moreover, traditionalist ideals of gender classify gender differences as biologically inevitable (e.g., men are perceived as innately more skilled at football, due to it being a masculine sport), with past research demonstrating that athlete quality (which often equates to increased entertainment) is an antecedent to attitudes towards women in sport (Mumcu & Marley, 2017). This further explains the finding that those with traditional ideals of gender possess less favourable AFLW consumption attitudes, as the presumption that AFLW is comparatively less entertaining, despite their elite status, may discourage engaging in the consumerism of the AFLW. These findings are similar to research from McCabe (2007), who found that those with more traditional gender ideology held more negative attitudes towards women’s basketball (i.e., a sport that has been historically male dominated), whilst those with egalitarian ideals of gender roles possessed more positive attitudes. This finding suggests that those with more progressive values, may be more likely to support and invest in women’s sport regardless of whether the sport is more traditionally associated with men. Previous literature also suggests that highly masculinised sports (i.e., contact and strength-based sports like football), such as rugby (Fallon & Jome, 2007), and weightlifting (Stepanova et al., 2018), are considered inappropriate for women, as these require women athletes to possess and engage with physicality that contradicts the binary of traditional gender roles. This perspective is also emphasised through the finding that attitudes towards women in traditionally feminine sports, or sports that require grace over physicality (e.g., gymnastics, dancing) are generally more favourable (Plaza et al., 2017; Ross & Shinew, 2008); further demonstrating a relationship between gender role ideology and attitudes towards women’s sport.

The results also highlight the importance of considering the sexualisation of female athletes in regard to attitude formation, coinciding with previous research that found that the general public had more negative attitudes towards women athletes who had been objectified by the media (Daniels et al., 2020). Sexual objectification enforces greater emphasis on an individual’s physical attributes, rather than attributes based on their abilities. Therefore, when women athletes are objectified, their identity is diminished and their competency as athletes is disregarded (Szymanski et al., 2010), further contributing the perception of women are inferior athletes. A significant correlation was present between gender role attitudes and attitudes towards the sexualisation of women, indicative of an increased likelihood of sexualising women athletes, for those with traditional gender ideology. This finding can be further explained by research suggesting that the sexual objectification of women is more prevalent in environments dominated by men and traditional gender ideology (Szymanski et al., 2010), such as the sporting industry (George et al., 2001), and more specifically, Australian football. Therefore, the AFLW exists in an environment in which the sexual objectification of women athletes may proliferate, potentially contributing to adverse perceptions of the league, as sexualised athletes evoke more negative consumer attitudes.

In addition to the impact of gender role attitudes and the sexualisation of women, other important factors emerged from the findings. Age and gender were found to be negatively associated with attitudes towards gender equality within the AFL, indicating that men (who were also found to be more likely to endorse traditional ideals of gender), and older adults, are less likely to endorse gender equality within the AFL. These findings are consistent with previous literature in other cultural contexts suggesting that men (Mumcu & Marley, 2017) and older adults (Mumcu et al., 2016) are less likely to have positive attitudes towards women’s sport.

As expected, increased knowledge of AFLW was positively associated with AFLW consumption attitudes, indicating those with more AFLW knowledge, also had more positive attitudes. Not only may those with more AFLW knowledge be more invested in the league, but they may also be more understanding of the inequalities faced by the AFLW, explaining this finding. However, attempts to increase public knowledge of, or exposure to the AFLW (e.g., through increased advertisement and airtime), may encourage more positive attitudes towards women’s football. Moreover, participants with a history of playing football, held more negative attitudes towards gender equality within the AFL, coinciding with previous findings that suggest former competitive athletes had less favourable attitudes towards women’s sports than non-athletes (Mumcu et al., 2016). It may be that those who play football feel more qualified to critique the performance of athletes within the sport (Mumcu et al., 2016). However, intercorrelations revealed that those with previous football experience were more likely to be men, and also more likely to endorse traditional gender role ideology, providing an additional explanation for this finding.

The relationship between sexual minority prejudice and negative attitudes towards gender equality within the AFL may be a result of the stereotypical conjecture that women in *masculine* sports are part of a sexual minority group (Scheadler & Wagstaff, 2018). Furthermore, the AFLW’s overt inclusion and support of sexual minority groups may further reinforce this stereotype, eliciting negative attitudes from those with more sexual minority prejudice, further explaining this finding. This may also be a consequence of traditional gender role ideology, as same-gender relationships are often seen as a violation of traditional ideals of masculinity and femininity, and therefore, are unacceptable to those with traditional ideals of gender (Nierman et al., 2007). This coincides with the relationship between traditional gender role ideology and negative attitudes towards women in sport, as sexualities beyond the heteronormative status quo are seen as a threat to societies in which patriarchy is vindicated by the traditional binary of gender roles (Nierman et al., 2007).

### Implications, limitations, and suggestions for future research

The current study provides unique insight into the understanding of factors impacting attitudes towards the AFLW. In order to promote the AFLW and contribute to closing the gap between the leagues, the implementation of several practical measures may encourage positive attitudes towards the AFLW. Firstly, the intervention of traditional gender role ideology could be implemented within advertising, through the normalisation of women’s participation in football. This could be achieved through exposure to scenarios that contradict stereotypes about the capabilities of women athletes, which may be beneficial in the promotion of gender equality within Australian football, therefore contributing to the improvement of these attitudes. Secondly, sports marketers could target their advertisements in a way that is more palatable to groups who have been found to be less supportive of the AFLW (e.g., men, and older adults), by presenting the AFLW in a manner that mirrors the coverage of men’s sports. This could include media reporting that focuses more on the style of play and match statistics, rather than a focus on individual players – which may lead to a potential improvement in attitudes and overall consumerism. However, given the dearth of research into the attitudes towards the AFLW, future research could further contextualise these findings, and capture underlying causes of negative attitudes towards the AFLW. By exploring these attitudes, researchers would be able to identify trends, and highlight unique perspectives, ultimately identifying how to proceed with intervention efforts against negative attitudes towards women’s participation in football in Australian society.

The unique contribution notwithstanding, it is important to recognise the limitations of this study. Firstly, the use of convenience sampling methodology may have reduced variability in participant responses. That is, most participants had average attitudes towards the AFLW, with only a small proportion displaying extremely negative attitudes (likewise, a small proportion displaying extremely positive attitudes). Secondly, convenience sampling may have also resulted in disproportionately within the demographics (e.g., a higher proportion of young people, women, and South Australian residents), indicating bias within the sample; making it difficult to generalise to the broader population of AFL supporters. Future research should aim to expand the sample size and participant demographics (e.g., age, gender, and location) to include a more balanced participation from the wider Australian public.

Third, due to correlational data, causation cannot be implied by the current findings. Identifying key areas of intervention is an important component of attitudinal research, and future research could use experimental methodologies to obtain a broader understanding of the factors contributing to attitudes towards the AFLW. This could be achieved by developing, on the basis of these findings, a targeted intervention for those with poorer gender role attitudes to improve attitudes towards the AFLW, and women in sport more generally.

## Conclusion

Despite the increased participation and involvement of women within the Australian football industry, the results of this study emphasise the existence of negative attitudes towards the AFLW, and potentially women’s sport more generally. These findings suggest that perceptions of football as a sport that is predominantly appropriate for men are still prevalent in Australian society, adding to the growing body of contemporary literature that suggests women’s participation in traditionally masculine sports is still perceived negatively. Furthermore, the results highlighted the importance of several factors in the prediction of attitudes towards the AFLW which contributes to our understanding of women’s sports consumerism in Australia more broadly. However, it is evident that further research is still needed to identify the underlying causes of these negative attitudes before intervention efforts can be developed. Understanding the predictors of negative attitudes towards women’s sport is central to challenging the gendered discourse within the football community and the sporting industry more generally, that serves to maintain the inequalities between women’s and men’s leagues.

## Data Availability

The de-identified dataset and materials associated with this article are available from the corresponding author upon reasonable request.
